# Features of non-traumatic spinal cord infarction on MRI: Changes over time

**DOI:** 10.1371/journal.pone.0274821

**Published:** 2022-09-22

**Authors:** Bo Ra Kim, Kyung Seok Park, Hyo Jin Kim, Jun Yup Kim, Bo Ram Kim, Eugene Lee, Joon Woo Lee

**Affiliations:** 1 Department of Radiology, Seoul National University Bundang Hospital, Seongnam, South Korea; 2 Department of Radiology, Dong-A University Hospital, Busan, South Korea; 3 Department of Neurology, Seoul National University Bundang Hospital, Seongnam, South Korea; 4 Department of Radiology, SMG-SNU Boramae Hospital, Seoul, South Korea; 5 Seoul National University College of Medicine, Seoul, South Korea; University of Milan-Bicocca, ITALY

## Abstract

**Background and purpose:**

Spinal cord infarction (SCI) is difficult to diagnosis using MRI findings. We aimed to suggest the optimal timing of MRI studies for diagnosing SCI.

**Materials and methods:**

This retrospective study was approved by our institutional review board. The requirement for informed consent was waived. MRI scans of SCI patients diagnosed between January 2015 and August 2019 were enrolled in the SCI group and subdivided according to the interval between symptom onset and time of MRI scan (A, within 6 h; B, 6–12 hours; C, 12–24 hours; D, 24–72 hours; E, 3–7 days). Three radiologists analyzed the T2WI scans and evaluated the confidence level of diagnosing SCI using a five-point Likert scale: 1, certainly not; 2, probably not; 3, equivocal; 4, probably yes; 5, certainly yes. Scores of 4 and 5 were defined as “T2WI-positive SCI” and scores of 1–3 were defined as “T2WI-negative SCI”.

**Results:**

The SCI group included 58 MRI scans of 34 patients (mean age, 60.6 ± 14.0 years; 18 women). The T2WI positivity rate was 72.4% (42/58). In contrast to the other subgroups, subgroup A included fewer cases of T2WI-positive SCI (1/4, 25%) than T2WI-negative SCI. A confidence score of 5 was the most common in subgroup D (4/27, 14.8%). Among the 12 patients who underwent MRI studies more than twice, confidence scores increased with time.

**Conclusion:**

In patients with suspected SCI showing equivocal initial MRI findings, follow-up MRI studies are helpful, especially when performed between 24 and 72 hours after symptom onset.

## Introduction

Spinal cord infarction (SCI) is a rare entity in comparison with cerebral infarction, accounting for only 1%–2% of all strokes [1–5]. Due to its rarity and nonspecific clinical manifestations, the diagnosis of SCI is challenging and more difficult in patients without risk factors such as prior aortic surgery [6,7]. Although treatment protocols for SCI are not well-established, early diagnosis and management are important to detect and correct the underlying causes, such as aortic dissection, as well as to prevent catastrophic neurological sequelae [6–10]. However, the diagnosis of SCI is challenging and depends on the identification of characteristic clinical features and compatible MRI findings and exclusion of alternative diagnoses. Zalewski NL et al. recently proposed diagnostic criteria and organized the MRI patterns of SCI (Table 1) [7].

**Table 1:** Proposed diagnostic criteria for spinal cord infarction (by Zalewski NL et al., 2019).

Criteria	
1	Acute nontraumatic myelopathy (no preceding progressive myelopathy)
	Onset to nadir severe deficits* 12 hours or less
	If stuttering course is more than 12 hours, severe deficits* rapidly develop 12 hours or less
2	Magnetic resonance imaging
	A No spinal cord compression
	B Supportive: intramedullary T2-hyperintense spinal cord lesion
	C Specific (1 of): diffusion-weighted imaging/apparent diffusion coefficient restriction
	associated vertebral body infarction
	arterial dissection/occlusion adjacent to lesion
3	Cerebrospinal fluid
	Noninflammatory (normal cell count, IgG index and no oligoclonal bands)
4	Alternative diagnoses
	Alternative diagnosis is not more likely
Type of spinal cord infarction (SCI)	
Definite spontaneous SCI: 1, 2A, 2B, 2C, 4	
Probable spontaneous SCI: 1, 2A, 2B, 3, 4	
Possible spontaneous SCI: 1, 4	
Definite periprocedural SCI: 1, 2A, 2B, 4	
Probable periprocedural SCI: 1, 4	

SCI, spinal cord infarction.

* A severe acute deficit (motor and/or sensory) typically consists of loss of antigravity strength or worse, and severe objective sensory loss impairing function (e.g., severe sensory ataxia).

Intramedullary hyperintense lesions on T2WI are important for the diagnosis of SCI. In our clinical experience, however, cord signal changes were equivocal in the early stage and became apparent in follow-up MRI evaluations. In the literature, few authors have focused on changes in the MRI features of SCI over time, and there is no consensus on the proper time for follow-up imaging examinations in cases with suspected SCI [6,7,11–13].

In this study, we hypothesized that the evolutionary changes in MRI findings in patients with SCI are relatively regular and predictable. Thus, our study aimed to analyze the serial MRI findings in SCI over time and to suggest an optimal timing of MRI examinations for correct diagnosis of SCI.

## Materials and methods

### Study population

**Fig 1:**
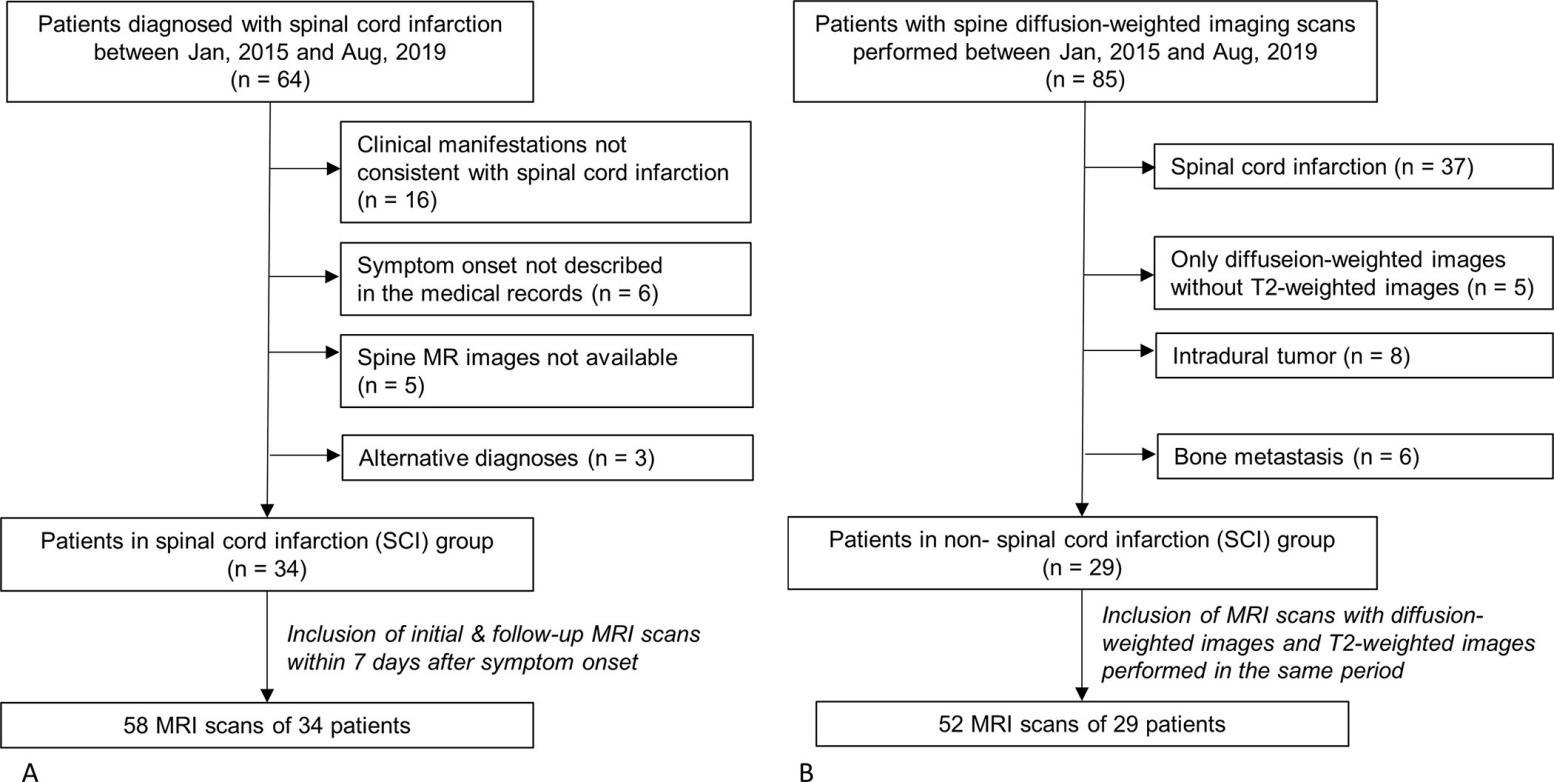
Flowchart of the study population recruitment. A, Spinal cord infarction (SCI) group. B, Non-spinal cord infarction (SCI) group.

This retrospective study was conducted in accordance with the tenets of the Declaration of Helsinki after obtaining approval from the institutional review board of Seoul National University Bundang Hospital (No.: B-2002-597-105). The requirement for informed consent was waived. To identify the study group, we performed searches using the diagnostic code for spinal cord infarction in our institutional electronic medical record system among cases recorded between January 2015 and August 2019. The neurologist (with 20 years of experience) subsequently reviewed the medical records of the patients to verify the diagnosis of SCI. Inclusion criteria were a final diagnosis of SCI with appropriate clinical (clear timeline and specificity of deficits, exclusion of alternative diagnoses, and/or the presence of causative events such as prior aortic surgery) and imaging data (MRI findings consistent with SCI and/or ruling out alterative diagnoses). The exclusion criteria were as follows: (a) clinical manifestations not consistent with SCI, such as trauma history and subacute or chronic symptoms; (b) symptom onset not described in the medical records; (c) spine MRI data not available; and (d) alternative diagnosis confirmed on clinical evaluations, including cerebrospinal fluid (CSF) studies. Initial MRI scans and follow-up MRI scans obtained within 7 days after symptom onset were included in the SCI group (Fig 1). To analyze the MRI features of SCI over time, MRI scans of the SCI group were subdivided according to the interval between symptom onset and the time of MRI scan as follows: (A) within 6 h, (B) between 6 and 12 h, (C) between 12 and 24 h, (D) between 24 and 72 h, and (E) between 3 and 7 days.

For the control group, we searched for patients with spine DWI scans in our institutional picture archiving and communication system (PACS) between January 2015 and August 2019. Of these, (a) patients diagnosed with SCI, (b) those with only DWI without T2WI scans, (c) patients with intradural tumors, and (d) patients with bone metastases were excluded. MRI scans of the remaining patients were included in the non-SCI group.

### Image analysis

As we included MRI scans acquired from other institutions as well as our own, the scanned areas and imaging protocols were diverse. Axial and sagittal T2WI and sagittal DWI scans were used for image analysis. Image analysis was performed in two discrete sessions. In the first session, three radiologists (two with two years of experience each and one with seven years of experience) independently reviewed the MRI scans. The reviewers were blinded to the group assignment as well as the clinical or laboratory information of the patients. They analyzed T2WI scans and evaluated the confidence scores for diagnosing SCI. In patients with DWI scans, the usefulness of DWI for diagnosing or excluding SCI was also evaluated. The confidence of the decision was evaluated with a five-point Likert scale as follows: 1 = certainly not; 2 = probably not; 3 = equivocal; 4 = probably yes; and 5 = certainly yes. To analyze the diagnostic performance of T2WI scans, confidence scores of 4 and 5 were defined as “T2WI-positive SCI” and scores of 1–3 were defined as “T2WI-negative SCI.”

In the second session, one radiologist with two years of experience analyzed the pattern of cord signal changes on axial T2WI scans in the SCI group as follows: owl eyes, anterior u/v, hologrey, dorsal white matter, ventral white matter, lateral white matter, and holocord. In complicated cases, image patterns were discussed with a senior radiologist (with 19 years of experience).

### Statistical analysis

The confidence scales for diagnosing SCI on T2WI and the usefulness of DWI in the SCI and non-SCI groups were compared using independent t-tests. In the subgroup analysis of the SCI group, the differences in the confidence scores for diagnosing SCI on T2WI and the usefulness of DWI among the subgroups were analyzed using the Kruskal-Wallis test. To analyze the diagnostic performance of T2WI over time, Pearson’s chi-square test was used for comparison. Inter-reader reliability was evaluated using Kendell’s W test. The level of significance was set at p < 0.05, and all statistical analyses were performed using software (SPSS, version 20.0. Armonk, NY: IBM Corp.).

## Results

### Study population

For the study group, a total of 64 patients were identified from our institutional electronic medical record system. Of these, 30 patients were excluded on the basis of the following criteria: (a) clinical manifestations not consistent with SCI, such as trauma history and subacute or chronic symptoms (n = 16); (b) symptom onset not described in the medical records (n = 6); (c) spine MRI data not available (n = 5); and (d) alternative diagnosis confirmed on clinical evaluations, including cerebrospinal fluid (CSF) studies (n = 3; a 68-year-old man with spinal dural arteriovenous fistula, a 36-year-old woman with neuromyelitis optica, and an 8-year-old boy with acute transverse myelitis). Fifty-eight MRI scans of the remaining 34 patients (mean age, 60.6 ± 14.0 years; 18 women) were enrolled in the SCI group (Table 2). Among them, four MRI scans (including one DWI scan) were acquired within 6 h (subgroup A); five (including one DWI scan), between 6 and 12 h (subgroup B); seven (including one DWI scan), between 12 and 24 h (subgroup C); 27 (including 14 DWI scans), between 24 and 72 h (subgroup D); and 15 (including 12 DWI scans), between 3 and 7 days (subgroup E) after symptom onset. The non-SCI group included 52 MRI scans of 29 patients (mean age, 60.0 ± 18.8 years; 10 women). Most of the patients were clinically diagnosed with demyelinating diseases, such as neuromyelitis optica, multiple sclerosis, or idiopathic transverse myelitis.

**Table 2:** Sample size and demographics of the spinal cord infarction group and non-spinal cord infarction group.

		SCI group	non-SCI group
The number of patients		34	29
The number of MR scans		58	52
Age (mean ± standard deviation)		60.6 ± 14.0	60.0 ± 18.8
Sex	male	16 (47.1%)	19 (65.5%)
	female	18 (52.9%)	10 (34.5%)

SCI, spinal cord infarction.

### Image analysis

The confidence scores for diagnosing SCI on T2WI in the SCI and non-SCI groups were significantly different (p < .001) and were higher in the SCI group. The confidence scores for the usefulness of DWI for diagnosing or excluding SCI were also significantly higher (p < .001) in the SCI group. The rates of T2WI positivity were 72.4% (42/58) in the SCI group and 0% in the non-SCI group.

In the subgroup analysis, the confidence scores for diagnosing SCI on T2WI (p = .214) and the usefulness of DWI (p = .669) were not significantly different among the subgroups. Differences in diagnostic performance (T2WI-positivity and T2WI-negativity) were also not significantly different (p = .083) among the subgroups. However, T2WI-positivity was observed in 25%, 60%, 71.4%, 70.4%, and 93.3% of the cases in subgroups A, B, C, D, and E, respectively (Table 3). A confidence score of 5 was the most common in subgroup D.

**Table 3:** The confidence scores for diagnosing spinal cord infarction on T2WI, the rate of T2WI-positive spinal cord infarction, and the types of SCI in each subgroup.

		Subgroup A (within 6 hours)	Subgroup B (6–12 hours)	Subgroup C (12–24 hours)	Subgroup D (24–72 hours)	Subgroup E (3–7 days)
Confidence scale of diagnosing SCI on T2WI*	1	0	0	1	0	1
	2	0	2	0	2	0
	3	3	0	1	6	0
	4	1	3	4	15	12
	5	0	0	1	4	2
MR-positive SCI†		1/4 (25%)	3/5 (60%)	5/7 (71.4%)	19/27 (70.4%)	14/15 (93.3%)
Types of SCI‡	Definite spontaneous SCI	0/4 (0%)	1/5 (20%)	1/7 (14.3%)	11/27 (40.7%)	11/15 (73.3%)
	Probable spontaneous SCI	1/4 (25%)	2/5 (40%)	4/7 (57.1%)	8/27 (29.6%)	3/15 (20%)
	Possible spontaneous SCI	3/4 (75%)	2/5 (40%)	2 (28.6%)	8/27 (29.6%)	1/15 (6.7%)

* The confidence scales of the decision was evaluated with a five-point Likert scale: 1, certainly not; 2, probably not.

3, equivocal; 4, probably yes; 5, certainly yes.

† The confidence scales of 4 and 5.

‡ The types of SCI are divided according to the diagnoatic criteria proposed by Zalewski NL et al. There was no periprocedural SCI.

Types of SCI can be divided according to the clinical feature and MRI findings, as mentioned in Table 1. There was no periprocedural SCI, and the types of SCI in each subgroup are shown in Table 3. The proportion of definite SCI increased with time, and the proportion of possible SCI decreased with time.

**Fig 2:**
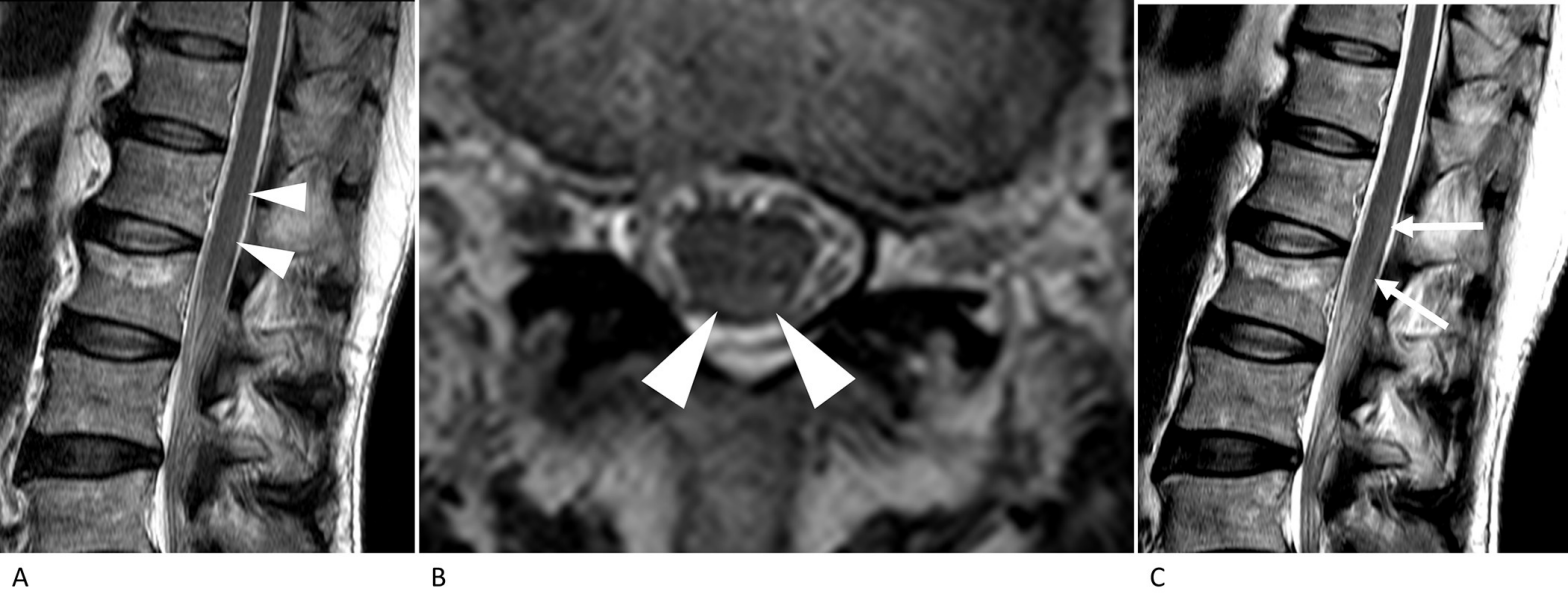
T2-weighted MRI of the thoracolumbar spine in the patient with spinal cord infarction (76-year-old man, patient number 1 in Table 4). On the sagittal (A) and axial (B) images obtained between 12 and 24 h after symptom onset, mild hyperintensity is suspected at the dorsal spinal cord (arrowheads). The authors evaluated the confidence score to be 3. Follow-up MRI (C) performed between 24 and 72 h after symptom onset shows pencil-like hyperintensity at the dorsal aspect of the spinal cord (arrows). The authors evaluated the confidence score to be 4.

**Fig 3:**
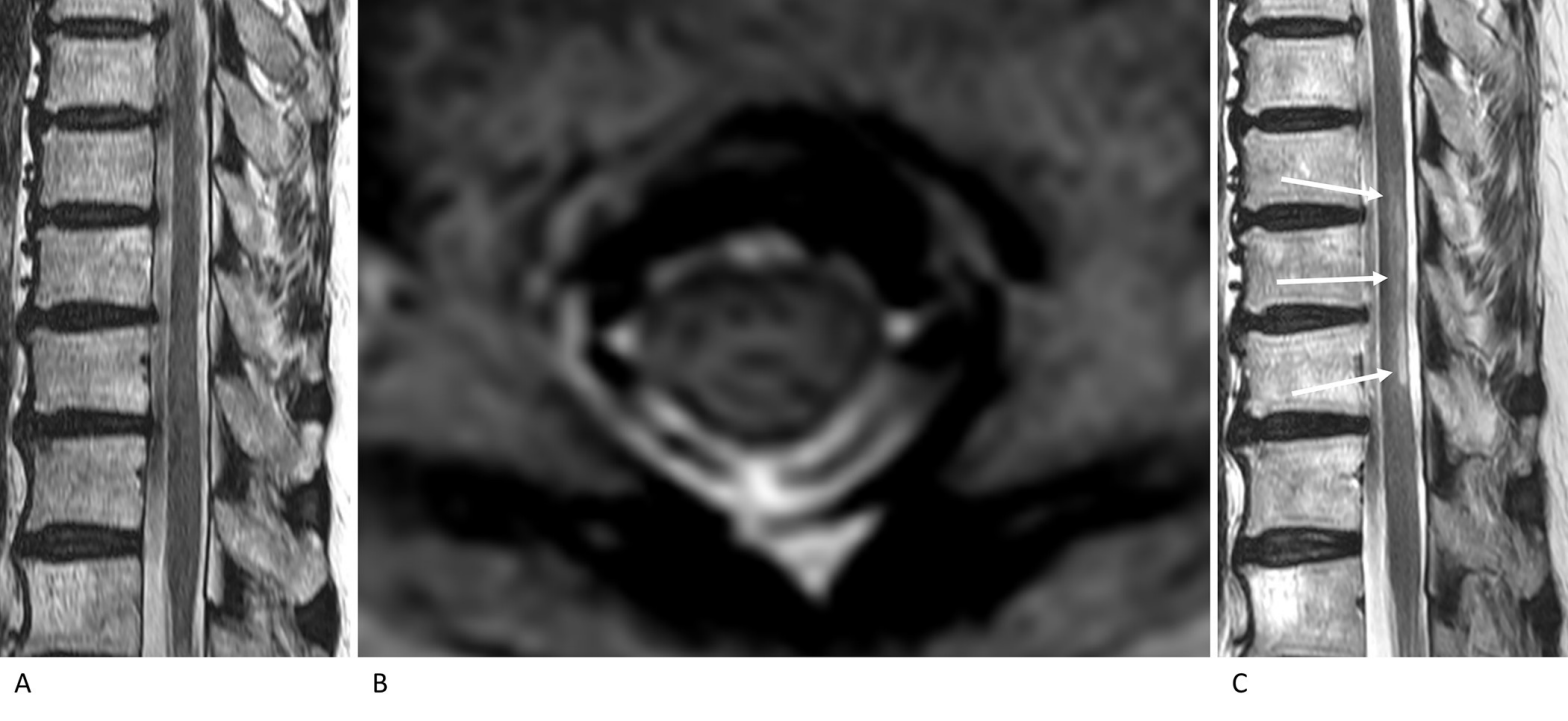
T2-weighted MRI of the lower thoracic spine in a patient with spinal cord infarction (80-year-old woman, patient number 9 in Table 4). On sagittal (A) and axial (B) images obtained between 6 and 12 h after symptom onset, changes in the cord signal are not obvious. The authors evaluated the confidence score to be 2. Follow-up MRI (C) performed between 24 and 72 h after symptom onset shows distinct hyperintensity in the dorsal spinal cord (arrows). The authors evaluated the confidence score to be 4.

In the SCI group, 12 patients had undergone MRI studies more than twice at different times, corresponding to the different subgroups (Table 4). Changes in the confidence scores for diagnosing SCI on T2WI between the initial and follow-up scans were noted in nine patients. Of these, four patients showed T2WI-negativity in the initial study and T2WI-positivity in the follow-up study: one patient showed scores of 3 between 12 and 24 h and 4 between 24 and 72 h (Fig 2); the second showed scores of 3 within 6 h and 4 between 6 and 12 h; the third showed scores of 2 between 24 and 72 h and 4 between 3 and 7 days; and the fourth showed a score of 2 between 6 and 12 h and 4 between 24 and 72 h (Fig 3). The confidence scores mostly increased over time in the same patients with SCI.

**Table 4:** The confidence scores for diagnosing spinal cord infarction on T2WI in patients of different spinal cord infarction subgroups who underwent MRI scans at more than two different time points.

patient number	age/sex	confidence scores of diagnosing spinal cord infarction on T2-weighted images*				
		Subgroup A (within 6 hours)	Subgroup B (6–12 hours)	Subgroup C (12–24 hours)	Subgroup D (24–72 hours)	Subgroup E (3–7 days)
1	76/M			3	4	
2	58/F	4		4		
3	55/M	3	4			
4	58/F				2	4
5	63/M		4		4	
6	73/M				5	4
7	46/F			1	3	
8	36/F	3			3	1
9	80/F		2		4	
10	64/M			4	5	4
11	60/M		4		5	
12	80/F				4	4

M, male; F, female.

*The confidence scores for the decision were evaluated with the following five-point Likert scale: 1, certainly not; 2, probably not; 3, equivocal; 4, probably yes; 5, certainly yes.

Kendall’s W coefficients were 0.8402 (p < .001) for the confidence score of diagnosing SCI on T2WI, and 0.6404 (p < .001) for the confidence score of the usefulness of DWI.

In the pattern analysis of cord signal changes in the SCI group, owl eyes, anterior u/v, hologrey pattern, dorsal white matter involvement, ventral white matter involvement, lateral white mater involvement, and holocord involvement were observed in 9, 4, 21, 12, 1, 3, and 7 cases, respectively. Fourteen cases showed more than two patterns simultaneously. One patient showed five different patterns (owl eyes, anterior u/v, hologrey pattern, dorsal white matter involvement, and holocord involvement), observed at different levels of the spinal cord.

## Discussion

Subgroup A included more cases of T2-weighted imaging (T2WI)-negative spinal cord infarction (SCIs) than T2WI-positive SCIs. The rates of T2WI-positivity increased with time; however, the confidence score of 5 (certainly yes) for diagnosing SCI on T2WI was the most common in subgroup D (4/27, 14.8%), in which assessments were performed between 24 and 72 hours after symptom onset. Among the 12 SCI patients in Table 4, two presented with T2WI-positivity on the follow-up MRI scan between 24 and 72 hours. In three of the 12 patients, the confidence scores decreased on follow-up MRI scans and were the highest between 24 and 72 hours. Therefore, SCI may present with equivocal findings on initial MRI scans, especially within 6 h after symptom onset. In these cases, follow-up MRI is recommended between 24 and 72 h, and the addition of a diffusion-weighted sequence is not necessary.

Changes in the MRI findings in cerebral infarction have been demonstrated in the literature [14]. In early hyperacute cerebral infarction within 6 hours of symptom onset, apparent diffusion coefficient maps and DWI may show positive findings. Positive FLAIR image findings can be observed 6–12 hours after symptom onset. Perfusion MRI is useful in diagnosing and determining treatment options for cerebral infarction [15].

On the other hand, a few reports have described the evolution of MRI findings in SCI with a small number of patients without a control group. Alblas CL et al. evaluated five SCI cases and reported that MRI features may show predictable changes over time in SCI [13]. In their article, MRI showed negative findings in the acute phase, T2 abnormalities over several days, and gadolinium enhancement subsequently. In other studies, authors reported negative MRI findings in the initial study and positive findings on repeated MRI in SCI patients [6,7,11]. These results are consistent with our findings. However, the optimal time for follow-up imaging has not been evaluated in previous studies. Kuker et al. reported the T2WI and DWI features in three patients with SCI [12]. On the initial MRI scans performed within 30 hours after symptom onset, both T2WI and DWI showed positive findings. Repeated MRI in one week showed positive findings on T2WI and pseudonormalization on DWI.

The use of DWI in diagnosing SCI has been reported in the literature since 2000 [5,16,17]. As in cerebral infarction, several reports have described abnormal DWI findings in the early phase after symptom onset, with the shortest time interval of three hours [5,17]. However, there are several limitations to the use of DWI for evaluation of the spinal cord [5,7,13,17,18]. Due to the inherent motion sensitivity of DWI, the motion of the surrounding cerebrospinal fluid may cause gross artifacts. The limited spatial resolution of DWI also makes it difficult to image the small-sized spinal cord, and susceptibility artifacts may occur from the multiple interfaces of the bone and soft tissue in spinal DWI. Therefore, we believe that follow-up T2WI may be more useful than DWI for the diagnosis of SCI. Consequently, in our institutions, we routinely performed follow-up T2WI with DWI in cases of clinically suspected SCI.

Our study had several limitations. First, cases with other types of myelopathy may have been included in the SCI group. Although diagnostic criteria for SCI have been proposed, they have not yet been validated in other cohorts. Thus, diagnosis of spontaneous SCI remains difficult, and not all patients could undergo a complete diagnostic workup. Second, the sample size was small, particularly in the subgroups. However, because SCI is a rare disease entity, studies involving a large number of patients are very difficult. Third, the MRI protocols and scanned areas were diverse, and MRI scans from other institutes were included. This could affect the imaging interpretation, because subtle signal changes in the small-sized spinal cord might be under- or over-evaluated. Nevertheless, this study is meaningful in that it included different MRI scans from several different medical institutes. Fourth, the time intervals used to divide the subgroups were arbitrarily assigned. Considering the importance of early diagnosis, the time intervals were smaller in the early phase (6 h in subgroups A and B) and larger subsequently (12 h in subgroup C, 48 h in subgroup D, and 4 days in subgroup E). However, consensus and validation for the definitions of acuity and chronicity of SCI are required in the future. Fifth, there is lack of histopathologic evaluation that support the results of this study. In cerebral infarction, DWI and ADC map show positive finding in hyperacute stage within 6 hours, due to cytotoxic edema [14]. Hyperintensity on FLAIR image and T2WI is evident in 6 to 8 hours after symptom onset [14]. These were similar to SCI in this study, in that negative of equivocal T2WI findings were more common within 6 hours after symptom onset and the confidence score for diagnosing SCI mostly increased with time. However, the sample size was small and the number of DWI scans was small in this study. Histopathologic study and MRI study using various pulse sequences might be necessary in the future.

## Conclusion

In cases with clinical suspicion of SCI showing negative or equivocal MRI findings within less than 6 h after symptom onset, follow-up MRI studies are helpful, especially when they are conducted between 24 and 72 h.

## Supporting information

S1 Table The confidence scores for diagnosing spinal cord infarction on T2WI. (DOCX)

S2 Table The confidence scores of usefulness of DWI for diagnosing spinal cord infarction. (DOCX)
