# Supplementary material for: Features of non-traumatic spinal cord infarction on MRI: Changes over time
Source: PLoS One. 2022 Sep 22;17(9):e0274821. doi: 10.1371/journal.pone.0274821 (PMC9499193; doi:10.1371/journal.pone.0274821)
Supplement: S1 Table — (DOCX) [file pone.0274821.s001.docx]

**Table 1. The confidence scores for diagnosing spinal cord infarction on T2WI**

| **Group** | **Age** | **Sex** | **Interval between  symptom onset  and time of  MRI scan** | **Subgroup** | **Reader 1** | **Reader 2** | **Reader 3** |
| --- | --- | --- | --- | --- | --- | --- | --- |
| SCI | 56 | F | 21.5hr | C | 2 | 1 | 5 |
| SCI | 57 | F | 48hr | D | 4 | 2 | 3 |
| SCI | 76 | M | 19hr | C | 5 | 4 | 3 |
| SCI | 76 | M | 41.5hr | D | 5 | 5 | 4 |
| SCI | 58 | F | 3hr | A | 5 | 4 | 4 |
| SCI | 58 | F | 13hr | C | 5 | 4 | 4 |
| SCI | 55 | M | 3.5hr | A | 2 | 2 | 3 |
| SCI | 55 | M | 10hr | B | 4 | 3 | 4 |
| SCI | 61 | M | 4d | E | 5 | 5 | 4 |
| SCI | 69 | M | 5d | E | 5 | 5 | 5 |
| SCI | 58 | F | 26.5hr | D | 2 | 1 | 2 |
| SCI | 58 | F | 5d | E | 4 | 3 | 4 |
| SCI | 73 | F | 37hr | D | 5 | 4 | 5 |
| SCI | 68 | M | 52hr | D | 5 | 4 | 4 |
| SCI | 68 | M | 58hr | D | 4 | 4 | 4 |
| SCI | 56 | M | 35hr | D | 5 | 5 | 4 |
| SCI | 56 | F | 38hr | D | 5 | 3 | 4 |
| SCI | 56 | F | 66hr | D | 5 | 4 | 4 |
| SCI | 71 | M | 31hr | D | 5 | 4 | 3 |
| SCI | 55 | F | 28hr | D | 4 | 3 | 3 |
| SCI | 66 | F | 4d | E | 4 | 4 | 4 |
| SCI | 66 | F | 5d | E | 5 | 4 | 4 |
| SCI | 63 | M | 18hr | B | 5 | 3 | 4 |
| SCI | 63 | M | 34hr | D | 5 | 4 | 4 |
| SCI | 63 | M | 48hr | D | 5 | 4 | 4 |
| SCI | 73 | M | 48hr | D | 5 | 3 | 4 |
| SCI | 73 | M | 71hr | D | 5 | 3 | 5 |
| SCI | 73 | M | 5d | E | 5 | 4 | 4 |
| SCI | 60 | M | 4d | E | 5 | 4 | 4 |
| SCI | 71 | F | 4d | E | 5 | 4 | 5 |
| SCI | 29 | M | 10hr | B | 5 | 5 | 2 |
| SCI | 70 | F | 13hr | C | 5 | 4 | 4 |
| SCI | 70 | F | 22hr | C | 4 | 4 | 4 |
| SCI | 46 | F | 23hr | C | 2 | 2 | 1 |
| SCI | 46 | F | 36hr | D | 2 | 2 | 3 |
| SCI | 36 | F | 2hr | A | 2 | 2 | 3 |
| SCI | 36 | F | 31hr | D | 5 | 3 | 3 |
| SCI | 36 | F | 5d | E | 2 | 2 | 1 |
| SCI | 80 | F | 12hr | B | 1 | 2 | 2 |
| SCI | 80 | F | 66.5hr | D | 5 | 5 | 4 |
| SCI | 70 | F | 28.5hr | D | 5 | 4 | 4 |
| SCI | 70 | F | 36hr | D | 5 | 5 | 4 |
| SCI | 64 | M | 17hr | C | 5 | 5 | 4 |
| SCI | 64 | M | 31hr | D | 5 | 5 | 5 |
| SCI | 64 | M | 5d | E | 5 | 4 | 4 |
| SCI | 65 | F | 2-3hr | A | 3 | 2 | 3 |
| SCI | 11 | M | 6d | E | 3 | 4 | 4 |
| SCI | 60 | M | 8hr | B | 4 | 2 | 4 |
| SCI | 60 | M | 45hr | D | 5 | 5 | 5 |
| SCI | 80 | F | 57.5hr | D | 5 | 4 | 4 |
| SCI | 80 | F | 3.5d | E | 5 | 4 | 4 |
| SCI | 63 | F | 41hr | D | 4 | 3 | 4 |
| SCI | 63 | F | 45hr | D | 5 | 4 | 4 |
| SCI | 51 | F | 73hr | E | 4 | 4 | 4 |
| SCI | 70 | M | 3.5d | E | 5 | 3 | 4 |
| SCI | 70 | M | 4d | E | 5 | 5 | 4 |
| SCI | 62 | M | 35hr | D | 4 | 3 | 3 |
| SCI | 62 | M | 52hr | D | 4 | 4 | 2 |
| non-SCI | 44 | F |  |  | 2 | 3 | 1 |
| non-SCI | 44 | F |  |  | 1 | 3 | 1 |
| non-SCI | 44 | F |  |  | 2 | 2 | 2 |
| non-SCI | 50 | M |  |  | 1 | 1 | 1 |
| non-SCI | 50 | M |  |  | 1 | 1 | 1 |
| non-SCI | 21 | M |  |  | 4 | 5 | 1 |
| non-SCI | 21 | M |  |  | 2 | 4 | 2 |
| non-SCI | 23 | M |  |  | 2 | 3 | 1 |
| non-SCI | 23 | M |  |  | 1 | 2 | 1 |
| non-SCI | 55 | M |  |  | 3 | 1 | 1 |
| non-SCI | 55 | M |  |  | 1 | 1 | 2 |
| non-SCI | 39 | F |  |  | 2 | 2 | 1 |
| non-SCI | 39 | F |  |  | 2 | 2 | 2 |
| non-SCI | 76 | M |  |  | 2 | 1 | 1 |
| non-SCI | 76 | M |  |  | 2 | 1 | 1 |
| non-SCI | 53 | F |  |  | 4 | 3 | 1 |
| non-SCI | 53 | F |  |  | 1 | 2 | 1 |
| non-SCI | 66 | M |  |  | 2 | 2 | 1 |
| non-SCI | 66 | M |  |  | 2 | 2 | 2 |
| non-SCI | 64 | M |  |  | 2 | 2 | 2 |
| non-SCI | 75 | F |  |  | 1 | 1 | 1 |
| non-SCI | 24 | M |  |  | 1 | 1 | 1 |
| non-SCI | 85 | M |  |  | 1 | 2 | 2 |
| non-SCI | 85 | M |  |  | 1 | 1 | 1 |
| non-SCI | 81 | F |  |  | 1 | 1 | 1 |
| non-SCI | 72 | M |  |  | 1 | 2 | 1 |
| non-SCI | 55 | M |  |  | 4 | 1 | 1 |
| non-SCI | 70 | M |  |  | 2 | 1 | 1 |
| non-SCI | 81 | M |  |  | 2 | 2 | 1 |
| non-SCI | 48 | M |  |  | 5 | 1 | 1 |
| non-SCI | 77 | M |  |  | 2 | 3 | 2 |
| non-SCI | 77 | M |  |  | 5 | 3 | 3 |
| non-SCI | 56 | F |  |  | 1 | 1 | 2 |
| non-SCI | 72 | M |  |  | 2 | 1 | 2 |
| non-SCI | 72 | M |  |  | 1 | 1 | 1 |
| non-SCI | 73 | M |  |  | 2 | 2 | 1 |
| non-SCI | 73 | M |  |  | 2 | 2 | 2 |
| non-SCI | 52 | F |  |  | 1 | 1 | 1 |
| non-SCI | 52 | F |  |  | 1 | 1 | 1 |
| non-SCI | 34 | F |  |  | 2 | 2 | 2 |
| non-SCI | 34 | F |  |  | 3 | 2 | 2 |
| non-SCI | 34 | F |  |  | 2 | 2 | 2 |
| non-SCI | 60 | M |  |  | 4 | 3 | 2 |
| non-SCI | 60 | M |  |  | 4 | 2 | 2 |
| non-SCI | 70 | M |  |  | 2 | 2 | 2 |
| non-SCI | 70 | M |  |  | 2 | 2 | 2 |
| non-SCI | 86 | F |  |  | 1 | 1 | 1 |
| non-SCI | 86 | F |  |  | 2 | 2 | 1 |
| non-SCI | 77 | F |  |  | 1 | 2 | 1 |
| non-SCI | 77 | F |  |  | 1 | 2 | 1 |
